# Overview of evidence-based research on acupuncture for stroke treatment using magnetic resonance imaging technology

**DOI:** 10.3389/fnins.2024.1495435

**Published:** 2024-11-25

**Authors:** Chao Ke, Wenying Shi, Zhuo Zhou, Zhengrong Xie, Mengzi Sun, Juli Yu, Shengtao Shan, Wei Zhang

**Affiliations:** The First Hospital of Hunan University of Chinese Medicine, Changsha, China

**Keywords:** stroke, neuroimaging, acupuncture, magnetic resonance imaging, evidence map

## Abstract

**Background:**

Stroke is a neurological condition characterized by high rates of disability and mortality. Magnetic resonance imaging (MRI) is widely used to examine the mechanisms of acupuncture in stroke treatment.

**Purpose:**

This review provides neuroimaging evidence for the efficacy of acupuncture in treating stroke using MRI.

**Method:**

We conducted a comprehensive search of databases, including PubMed, Embase, Cochrane Library, China National Knowledge Infrastructure (CNKI), Wan Fang Data, Chinese BioMedical Literature Database (CBM), and Chonqing VIP (CQVIP), from inception to April 2024. Relevant neuroimaging studies on acupuncture for stroke were included, and the research findings were presented through charts and textual analyses.

**Results:**

A total of 158 studies were included, and the overall methodological quality of the included studies was moderate to high. The results were divided into three categories: basic characteristics, clinical characteristics, and quality assessment of the included literature.

**Conclusion:**

We elucidated the neural mechanisms underlying the effects of acupuncture on stroke; however, the evidence remains preliminary. There is a need for large-scale, well-designed, multimodal neuroimaging trials. This review represents the first active use of an evidence map to systematically review and illustrate the current state of neuroimaging research on the acupuncture treatment of stroke, thereby providing a valuable reference for future research.

## 1 Introduction

Globally, stroke has consistently been the leading cause of mortality and disability among individuals aged 50 and older (GBD Diseases and Injuries Collaborators, 2019), respectively, often resulting in varying degrees of neurological deficits (Benjamin et al., 2019). By 2050, over 200 million patients with stroke are expected to survive (Brainin et al., 2020), imposing a substantial financial burden on the healthcare system (Feigin et al., 2016; Katan and Luft, 2018). According to the Screening and Intervention Project for Individuals at High Risk of Stroke, the number of patients with stroke aged 40 and above in China has reached 12.42 million (Report on Stroke Prevention and Treatment in China Writing Group, 2023). Numerous scholars demonstrate the efficacy of acupuncture for stroke in high-impact academic journals (Zhang J. et al., 2023; Zhang J. S. et al., 2023; Zhang Y. et al., 2023). Clinical guidelines, including the Brazilian Practice Guidelines for Stroke Rehabilitation and Chinese Stroke Association Guidelines for Clinical management of Cerebrovascular Diseases, have integrated evidence-based recommendations for acupuncture in stroke management (Zhang et al., 2020; Minelli et al., 2022).

Abnormal variations in brain structure and function have been found in patients with stroke patients (Wang et al., 2019), suggesting its critical role in nerve function outcome and recovery. Consequently, assessing these post-stroke abnormalities and investigating the neural mechanisms of dysfunction is crucial for advancing treatment strategies and improving prognosis. In addition, exploring the mechanism of action of acupuncture can lay a solid theoretical foundation for the prevention and treatment of stroke by acupuncture, which is conducive to promoting the clinical application of acupuncture and has important clinical significance. The rapid development of neuroimaging, particularly magnetic resonance imaging (MRI), enables precise, non-invasive, and multimodal fusion to explore the pathophysiological mechanisms related to neuropsychiatric diseases. MRI technology has been widely used to identify functional and structural changes in the brain of patients with stroke and investigate acupuncture’s mechanism in treating stroke (Zhang J. et al., 2023; Zhang J. S. et al., 2023; Zhang Y. et al., 2023). We systematically reviewed MRI research on stroke treatments in recent years and used evidence mapping to provide clinical researchers with neuroimaging insight into acupuncture and stroke, offering new directions for future research.

## 2 Method

### 2.1 Search strategy

The electronic databases used for the systematic search from databases inception to 12 April 2024, included PubMed, Embase, Cochrane Database of Systematic Reviews (CDSR), Cochrane Controlled Trials Register (CENTRAL), China National Knowledge Infrastructure (CNKI), Chinese BioMedical Literature Database (CBM), Chonqing VIP (CQVIP), and Wanfang. Detailed search strategies are provided in Supplementary file S1.

### 2.2 Literature inclusion and exclusion criteria

The inclusion criteria were: (1) Study types: This review included clinical studies on acupuncture for stroke, limited to journal articles. (2) Participants: Study participants were diagnosed with stroke based on various diagnostic criteria. The study subjects underwent two MRI scans before or after acupuncture or one MRI scan during acupuncture. (3) Intervention types: The experimental group received acupuncture therapy alone or in combination with other conventional therapies. (4) Type of control: The control group evaluated the effects of acupuncture, with participants receiving sham acupuncture (SA), placebo acupuncture, no treatment, drugs, Chinese medicine, rehabilitation therapy, or other therapies. (5) Types of outcome: At least one MRI technique was used, resulting in MRI outcome measures. The exclusion criteria were: (1) Animal experiments, reviews, case studies, experience reports, or protocols; (2) Duplicate studies or data; (3) Studies where contacting the author via email did not resolve missing data or yield the necessary information.

### 2.3 Study selection and data extraction

Two authors (Chao Ke and Wenying Shi) independently screened and examined the features of all articles identified using the PICOS (population, interventions, comparators, outcomes, study design) selection criteria, and extracted the data for further evaluation. Subsequently, a cross-check was performed, and any disagreements were resolved through arbitration by a third author (Wei Zhang). Data extraction included the following: general study information (title, author, country, journal, journal level, year of publication, registration, and funding source); patient demographics (lesion location, first-episode, functional disorder category, stroke type, and disease stage); trial design (study type, total sample size, trial design, control design, and neuroimaging design); and outcome evaluation (MRI type and outcomes).

### 2.4 Data analysis and synthesis

The results were presented in a preferred reporting item for systematic reviews and meta-analyses (PRISMA) flow diagram using a combination of textual descriptions and charts, and visual representations were created using Microsoft Excel 2003, Original, and chiPlot website (https://www.chiplot.online/) for data analysis and synthesis. The presentation also included bubble plots, a fold line diagram, three-line table.

## 3 Result

**Figure 1:**
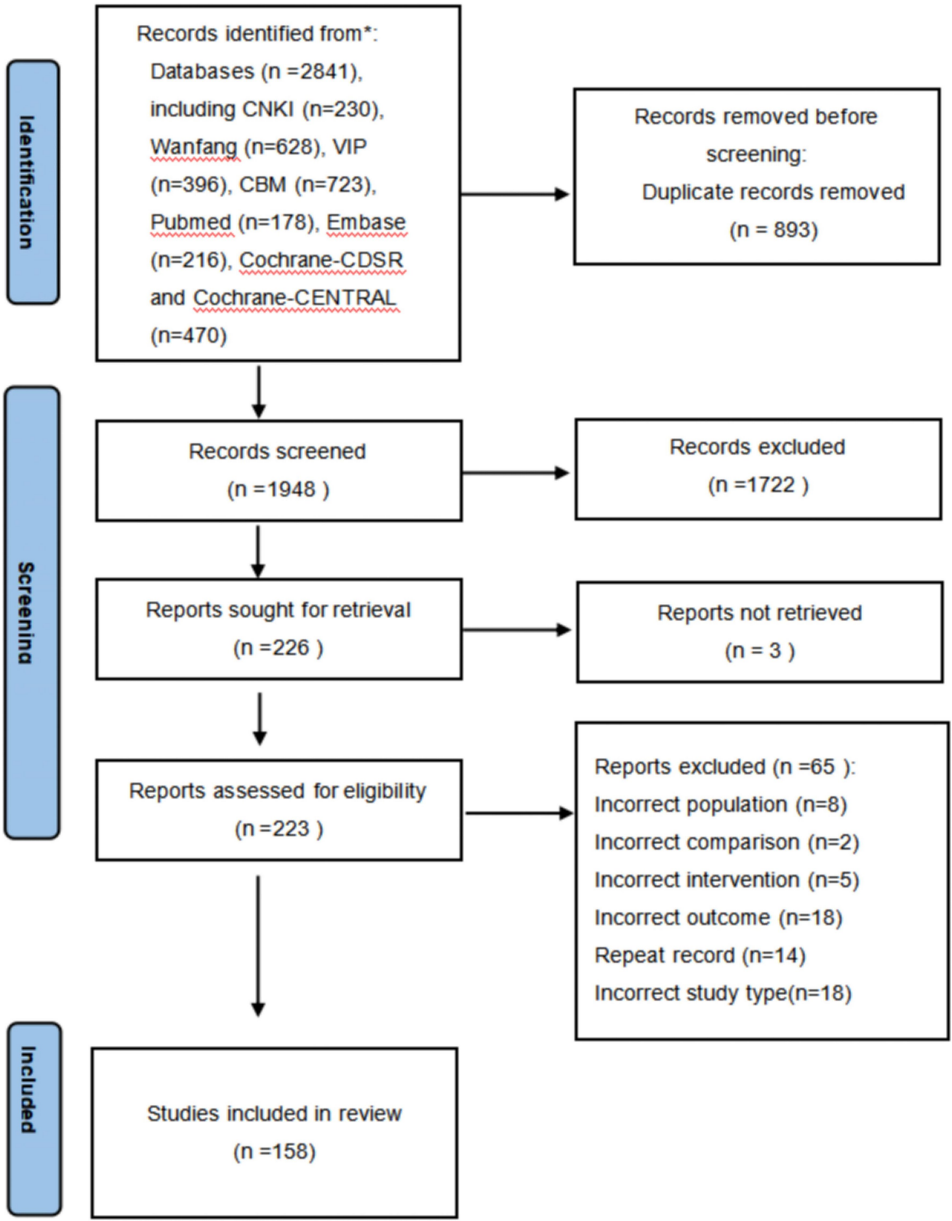
A PRISMA diagram of the literature selection.

A total of 2,841 literature items were retrieved using the search strategy. Initially, 1,948 articles remained after duplicates were removed. Subsequently, 1,722 records were excluded after reviewing the titles and abstracts. Three articles failed to obtain the full text. Finally, 158 studies were included in this meta-analysis. Details of the literature screening process are displayed in the PRISMA flowchart (Figure 1).

### 3.1 Basic characteristics of included studies

**Figure 2:**
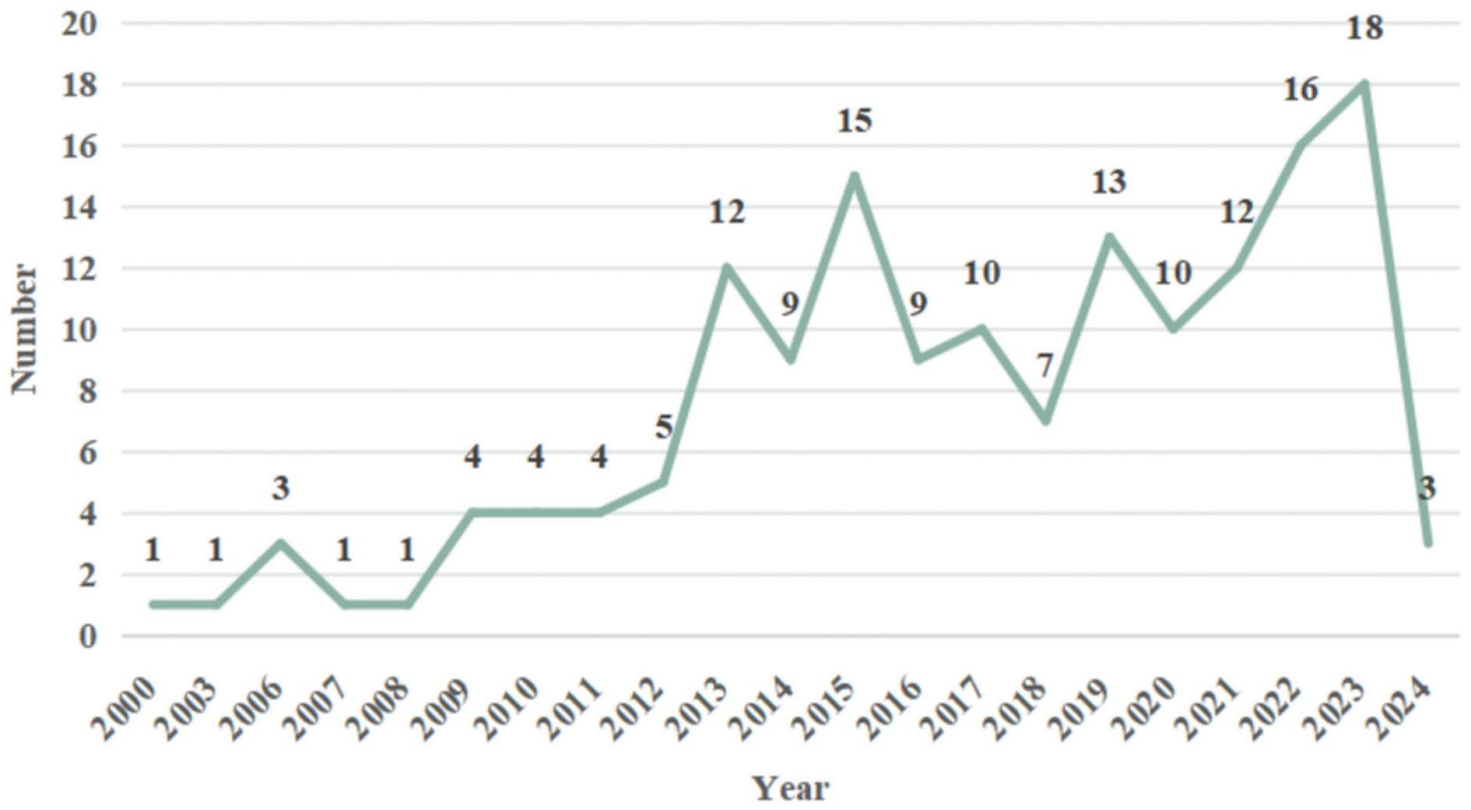
Annual publication trend of articles.

MRI studies on acupuncture for stroke began in 2,000, with 158 studies published between 2000 and April 2024. Results showed an increasing trend in research in recent years, peaking in 2023. The annual distribution of the literature is shown (Figure 2). A total of 158 studies were published across 96 distinct journals. Approximately 25.31% of the selected papers were published in the Science Citation Index Journal (SCI), 59.49% in core journals such as the Chinese Core Journal Criterion of Peking University (CSCD) and the China Core Periodicals of Science and Technology (CSTPCD), and the remaining 15.19% in other journals. Most studies received project funding (130/158, 82.28%), such as the Natural Science Foundation of China and 973 projects; very few studies reported registration status (13/158, 8.23%). Most studies were authored by Chinese authors, with only Germany and Boston contributing a single study each (Table 1).

**Table 1:** Basic characteristics of the included studies.

	*n* = 158
Country of authors	
China	156 (98.74%)
Boston	1 (0.63%)
Germany	1 (0.63%)
Journal grade	
1 Science Citation Index journal	40 (25.31%)
2 Chinese Science Citation Database journal	40 (25.31%)
3 Science and Technology/Peking University Core Journal	54 (34.18%)
4 Common journal	24 (15.19%)
Registered	
Yes	13 (8.23%)
Not reported	145 (91.77%)
Funding	
Yes	130 (82.28%)
Not reported	28 (17.72%)
Distribution of study types	
RCT	97 (61.4%)
Non-RCT	61 (38.6%)

### 3.2 Characteristics of the included studies

#### 3.2.1 Characteristics of the study population

The sample sizes of the 158 studies ranged from 5 to 200. Most studies (108, 68.35%) had sample sizes between 1 and 50. Of the studies, 46.2% reported that the patients recruited had a first-episode stroke. Concurrently, patients primarily had ischaemic stroke (134/158, 84.81%) and post-stroke motor impairment (80/158, 50.63%). Participants were mostly in the acute period and recovery period (112/158, 70.89%). The lesions were primarily distributed in the basal ganglia, corona radiata, and internal capsule. The results are presented in Table 2.

**Table 2:** People in the included studies.

	*n* = 158
Sample size	
1–50	108 (68.35%)
51–100	37 (23.42%)
101–200	13 (8.23%)
Whether first-episode stroke	
Yes	73 (46.2%)
No or not-mentioned	85 (53.8%)
Stroke type	
1 Ischemic stroke	134 (84.81%)
2 Haemorrhagic stroke	2 (1.27%)
3 Both stroke types or not-mentioned	22 (13.92%)
Post-stroke dysfunction	
1 Motor impairments	88 (55.7%)
2 Speech impairments	11 (6.96%)
3 Cognitive impairments	8 (5.06%)
4 Swallowing impairments	4 (2.53%)
5 sensory impairments	4 (2.53%)
6 Sleep impairments	1 (0.63%)
7 Mood impairments	1 (0.63%)
8 Shoulder-hand syndrome	1 (0.63%)
9 Both impairments (motor and cognitive Impairments, motor and sensory Impairments)	9 (5.7%)
10 Not-mentioned	31 (19.62%)
Clinical stage	
1 Acute period	29 (18.35%)
2 Recovery period	44 (27.85%)
3 Sequelae period	9 (5.7%)
4 Acute period to recovery period	39 (24.68%)
5 Recovery period to sequelae period	8 (5.06%)
6 Both periods	9 (5.7%)
7 Not-mentioned	20 (12.66%)
Location of lesions	
Mentioned	92 (58.23%)
Basal ganglia	61 (38.51%)
Internal capsule	11 (6.96%)
Corona radiata	30 (18.99%)
Not-mentioned	66 (41.77%)

#### 3.2.2 Characteristics of the intervention and the control

**Figure 3:**
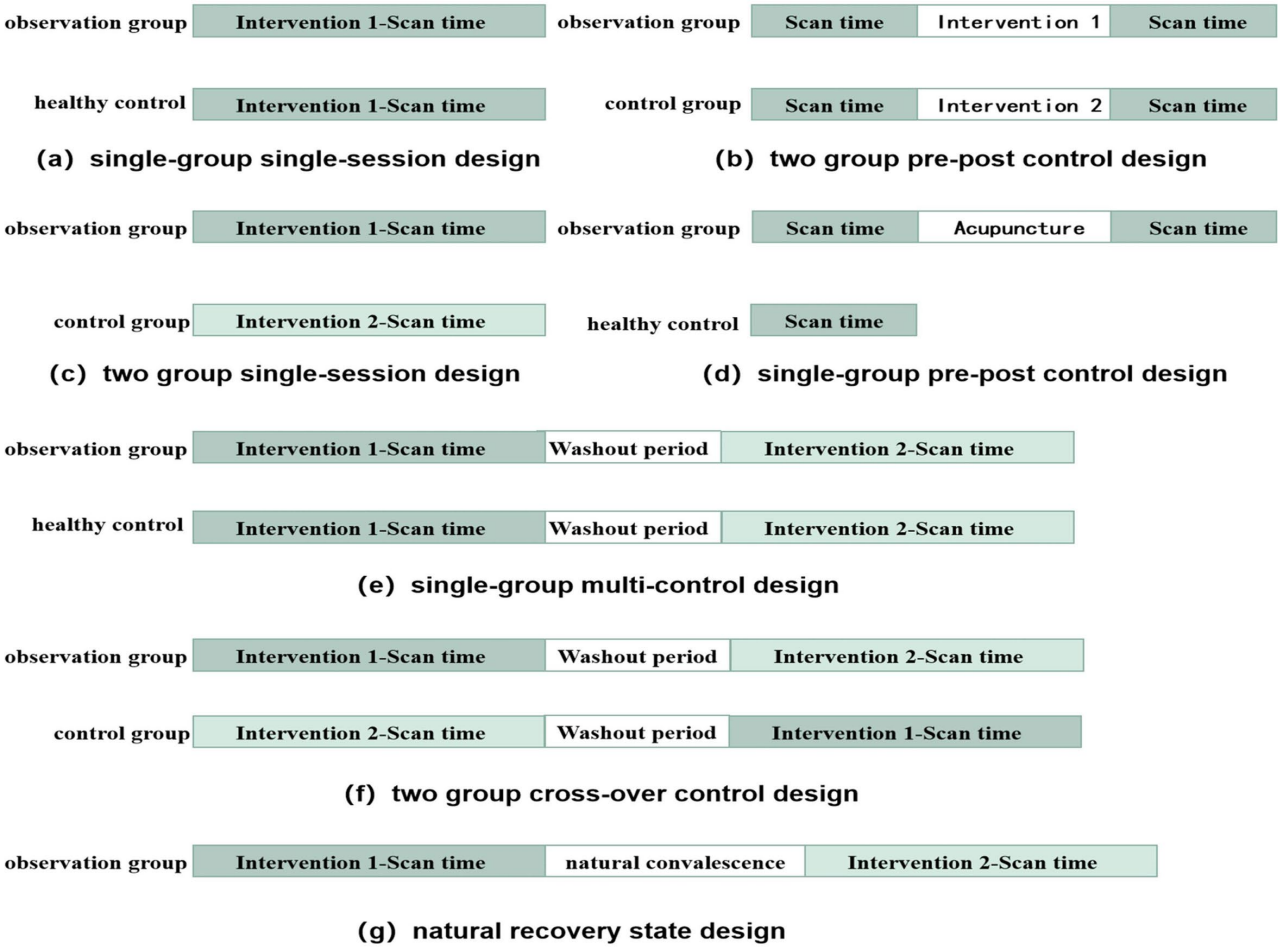
Trail design of the included studies.

**Figure 4:**
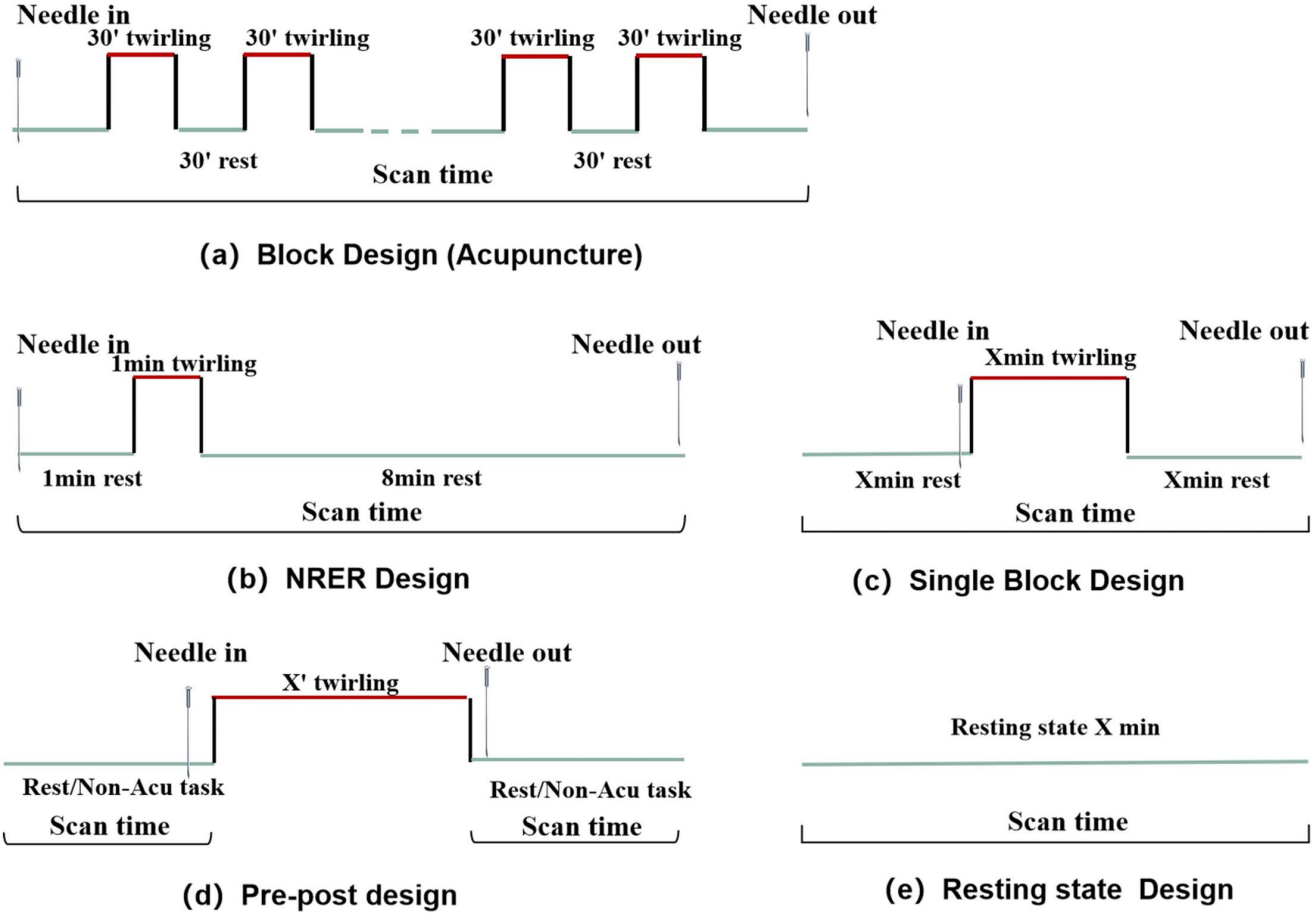
MRI Intervention design of the included studies.

We reviewed the MRI trial design for acupuncture in stroke studies, focusing on four commonly used methods: two-group pre–post control, single-group single-session, two-group single-session, and single-group pre-post control designs (Figure 3). Regarding MRI intervention design, most studies (85/158, 53.8%) used a resting-state mode, while the remaining (73/158, 46.2%) used a task-state mode. Among the task-state modes, 27 studies used the classical BLOCK design paradigm, 14 used the NRER paradigm, 9 used the MIX-BLOCK paradigm, 5 used the Pre-Post paradigm, and 3 used the Single-BLOCK paradigm (Figure 4).

**Figure 5:**
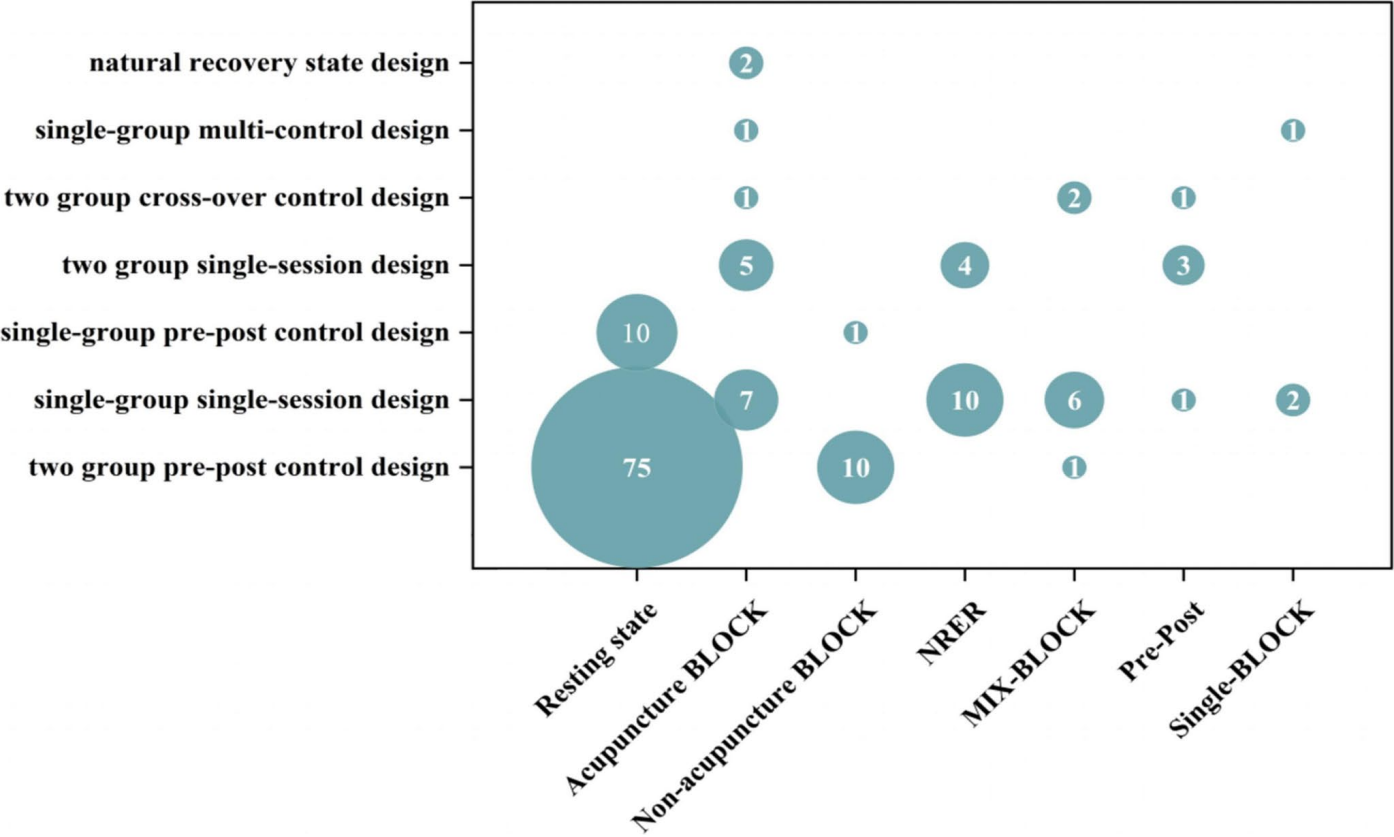
Bubble plots of trail design and MRI intervention design characteristics of the outcome.

Consequently, we created a bubble map illustrating the trial design and MRI intervention design (Figure 5). The most commonly used study protocol was the two pre-group control studies-resting-state mode observation. The control design was divided into two main purposes: mechanisms of acupuncture therapy and efficacy of acupuncture. The three most common control designs were: verum acupuncture combined with other therapies versus other therapies, verum acupuncture versus healthy control, and verum acupuncture versus SA. The outcomes are presented in Table 3.

**Table 3:** Trial, interventions, control design of the included studies.

	n = 158
Trial design	
4 two groups pre-post control design	88 (55.7%)
1 single-group single-session design	35 (22.15%)
2 two groups single-session design	15 (9.49%)
3 single-group pre-post control design	11 (6.96%)
5 two groups cross-over control design	5 (3.16%)
6 single-group multi-control design	2 (1.27%)
7 natural recovery state design	2 (1.27%)
MRI Intervention design	
Resting state	85 (53.8%)
Task state	73 (46.2%)
Classical BLOCK (Acupuncture)	16 (10.13%)
Classical BLOCK (Non-acupuncture)	11 (6.96%)
Non-repeated event-related (NRER)paradigm	14 (8.86%)
MIX-BLOCK paradigm	9 (5.7%)
Pre-post paradigm	5 (3.16%)
Single-BLOCK paradigm	3 (1.9%)
Mix-Single-BLOCK paradigm	2 (1.27%)
Other	13 (8.22%)
Control design	
Mechanisms of acupuncture therapy	153 (96.84%)
1 VA / VA combined with other therapies *VS* other therapies	77 (48.73%)
2 VA *VS* HC	46 (29.11%)
3 VA *VS* SA	20 (12.66%)
4 self-control before and after	14 (8.86%)
5 VA1 *VS* VA2	11 (6.96%)
6 Others (grouped according to disease course and condition)	4 (2.53%)
Efficacy of acupuncture	5 (3.16%)
Timing of the needle	1 (0.63%)
Needle-retention time	1 (0.63%)
Deqi and non-deqi	1 (0.63%)
Different acupoints	2 (1.27%)

In the control design, there are multi-arm tests; therefore, the number exceeds 158. VA, verum acupuncture; HC, healthy control; SA: Sham acupuncture.

**Figure 6:**
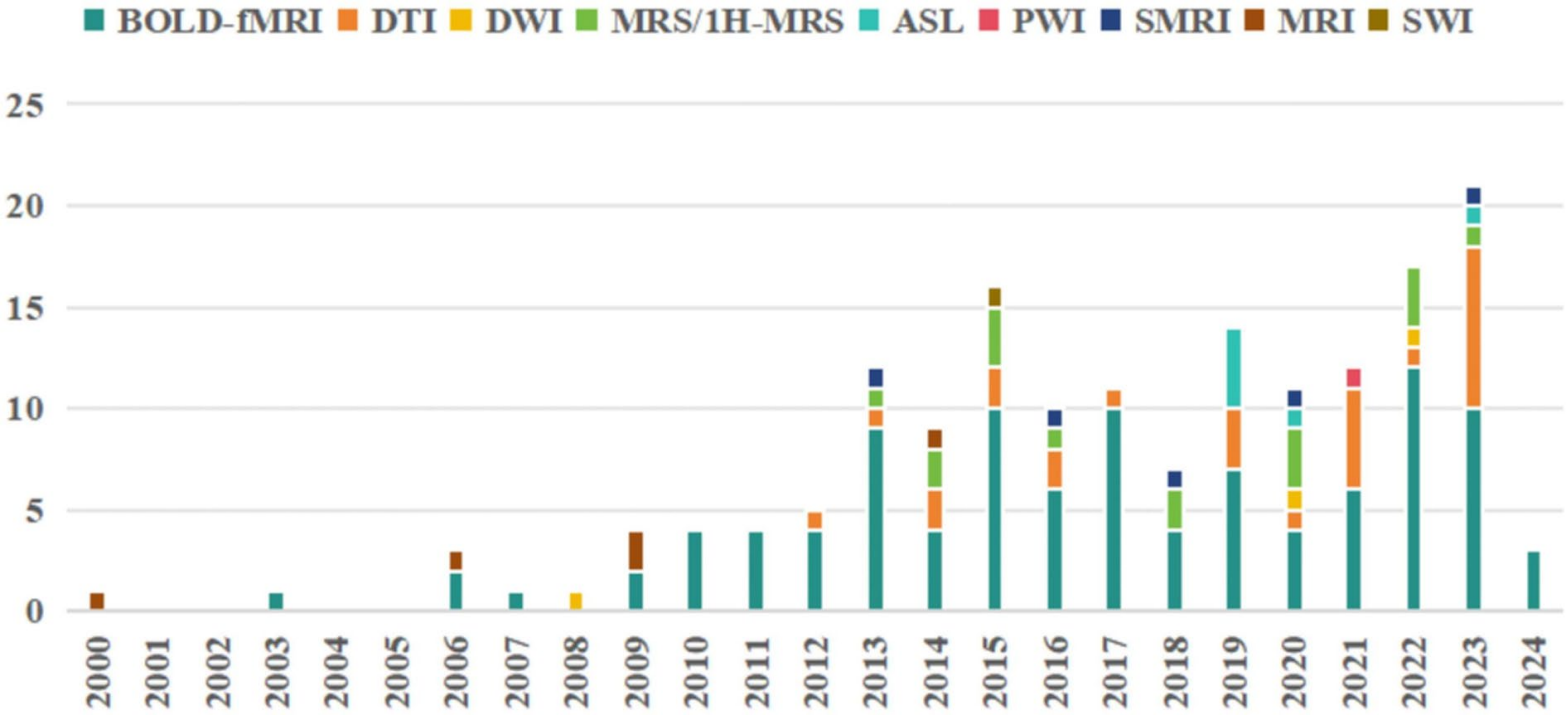
Annual frequency analysis chart of the MRI types.

**Figure 7:**
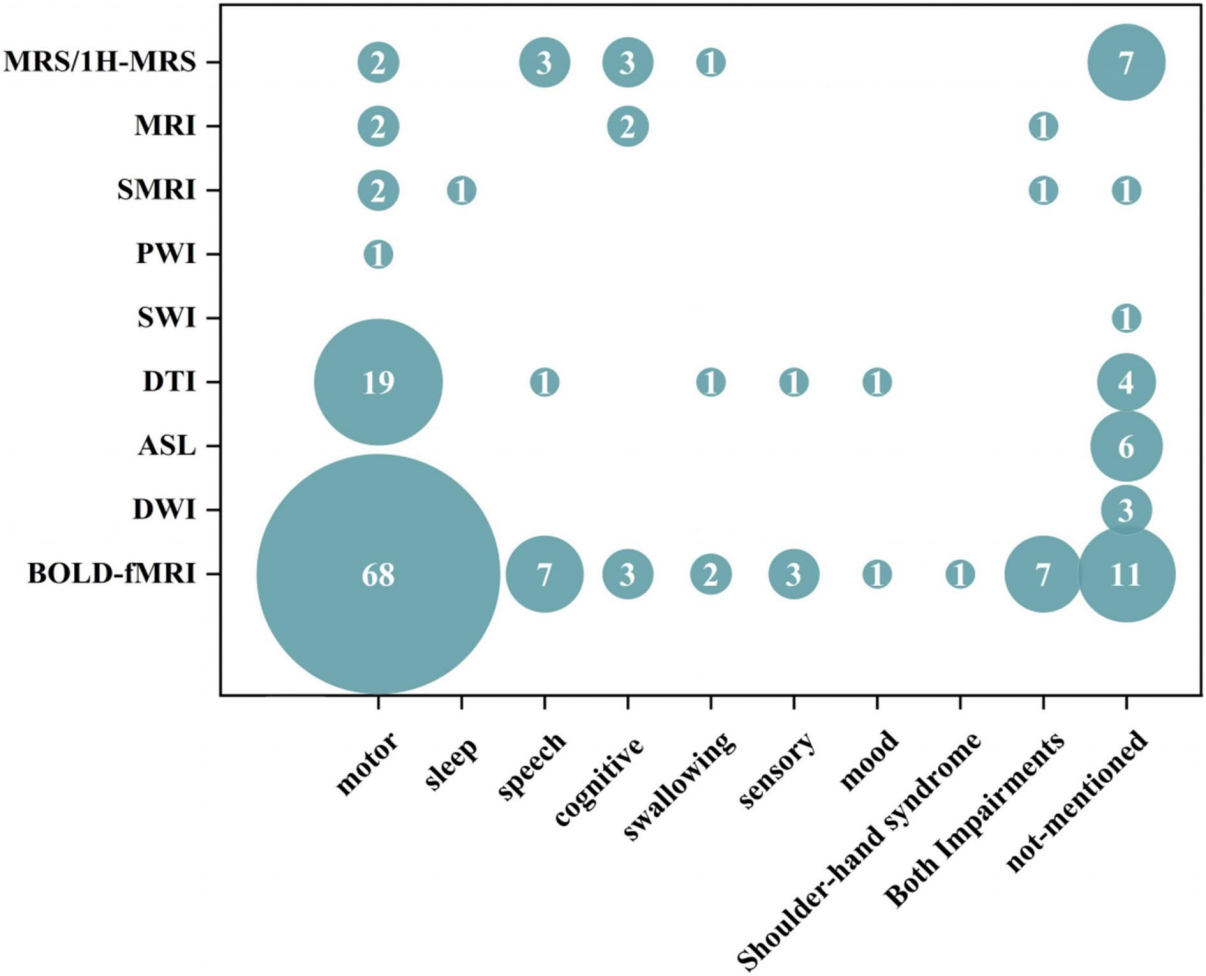
Bubble plots of each type of MRI and post-stroke dysfunction.

**Figure 8:**
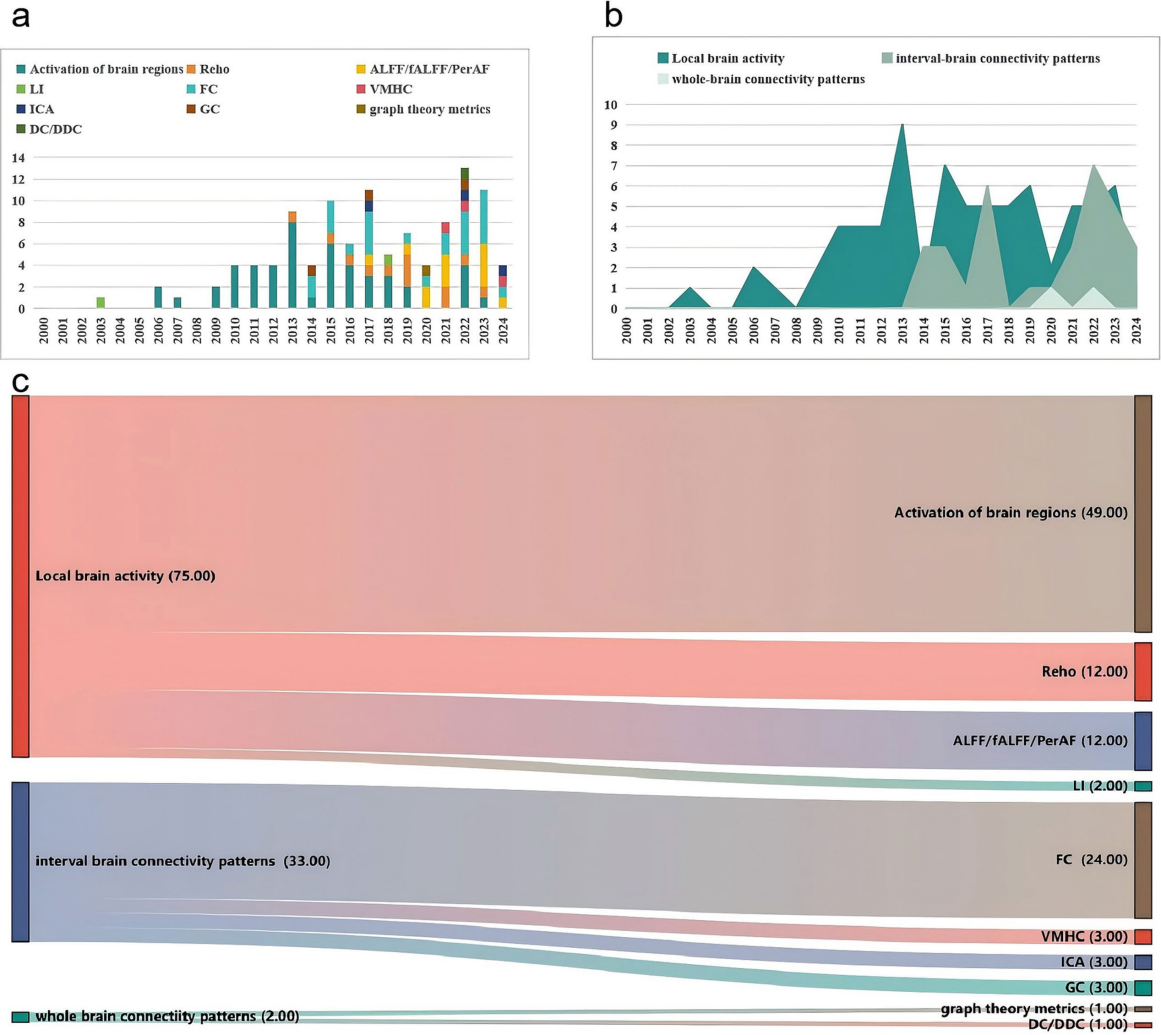
Frequency analysis chart of the efficacy of evaluation indices of BOLD-fMRI. **(a)** Annual frequency analysis chart of the efficacy of evaluation indices of BOLD-fMRI. **(b)** Mulberry fruit diagram of the frequency of evaluation indices of BOLD-fMRI (According to the three categories). **(c)** Mulberry fruit diagram of the frequency of evaluation indices of BOLD-fMRI.

Our results suggest that MRI of cerebral blood oxygen metabolism (104/158, 65.82%), diffusion MRI (30/158, 18.18%), magnetic resonance spectrum (16/158, 10.13%), perfusion MRI (7/158, 4.43%), and structural MRI (5/158, 3.16%) have been extensively used in acupuncture for stroke treatment. Researchers are increasingly preferring data collection using advanced MRI techniques. The detailed results are presented in Table 4. We conducted a comprehensive summary of the annual frequency chart for each type of MRI and post-stroke dysfunction (Figures 6, 7). The three most frequently used MRI modalities are blood-oxygen-level-dependent (BOLD)-functional MRI (BOLD fMRI), diffusion tensor imaging (DTI), and magnetic resonance spectroscopy/Proton-MRS (MRS/1H-MRS) (Figure 6). BOLD-fMRI has been in use since 2003, DTI since 2012, and MRS/1H-MRS since 2013. The three disorders under investigation were post-stroke motor, speech, and cognitive impairments (Figure 7). We further summarized the annual frequency maps of most BOLD fMRI indicators (Figure 8). The frequencies of brain activation areas, functional connectivity (FC), and regional homogeneity (Reho) have increased annually (Figure 8a). Since 2003, various indicators representing local brain activity, such as the activation of brain regions, Reho, amplitude of low-frequency fluctuation (ALFF)/fractional amplitude of low-frequency fluctuation (fALFF)/percent amplitude of fluctuation (PerAF) and lateralization index (LI) have emerged. Since 2013, the frequency of interval-brain connectivity patterns, such as FC, voxel-mirrored homotopic connectivity (VMHC), independent component analysis (ICA), and Granger causality (GC), has increased. Furthermore, since 2020, graph theory metrics, including degree centrality (DC), differential degree centrality (DDC), and other indicators representing whole-brain connectivity patterns, have been introduced (Figures 8b,c).

**Table 4:** Outcome characteristics of the included studies.

MRI of cerebral blood oxygen metabolism
*BOLD-fMRI*
Activation of brain regions(49), FC(24), ReHo(12), ALFF/fALFF/PerAF(12), ICA(3), LI(2), VMHC(3), GC (3), DC/DDC(1), graph theory metrics: clustering coefficient/small worldness/global efficiency/average local efficiency(1)
*SWI*
PV (1)
Diffusion MRI
*DTI*
FA/rFA(30), pyramidal tract condition (3), ADC(2), AD(2), RD(2), MD(1), rADC(1)
*DWI*
ADC(2), ischemic area(2)
*MRS/1H-MRS*
NAA(16),Cr(16),Cho(13),Lac(6),MI(1)
Perfusion MRI
*ASL*
CBF(6)
*PWI*
Blood supply (1)
*Structural MRI*
Grey matter voxel (5)
*Common MRI*
Lesions condition (3), width ectosylvian gyrus (2), the third ventricle width(2), mean sulcal width brain (2), frontal index (2), caudate index (2),lateral ventricles’ volume index(2)

FC: functional connectivity, ReHo: Regional homogeneity, ALFF: Amplitude of low frequency fluctuation, fALFF: fractional ALFF, ICA: Independent compo-nent analysis, VMHC: voxel-mirrored homotopic connectivity, GC: Granger causality, DC: Degree Centrality, DDC: Dynamic Degree Centrality, PerAF: percent amplitude of fluctuation, literality index: LI, PV: phase value, FA: Fractional Anisotropy, rFA: FA ratio, ADC: apparent diffusion coefficient, rADC: ADC ratio, AD: axial diffusivity, RD: radial diffusivity, MD: mean diffusivity, NAA: N-acetyl-aspartate, Cho: choline, Cr: creatine, Lac: lactate, MI: myo-inositol, CBF: cerebral blood flow. fMRI: functional MRI, DTI: diffusion tensor imaging, MRS: magnetic resonance spectroscopy, 1H-MRS: Proton MRS, DWI: diffusion weighted imaging, ASL: arterial spin labeling, PWI: perfusion weighted imaging, sMRI: Structural MRI. As for MRI types, there are multi-MRI tests, so the number exceeds 158.

## 4 Discussion

### 4.1 Included populations

Ischemic stroke accounts for approximately 87% of all stroke cases in the United States (Feigin et al., 2013) and 80% of all stroke cases in China (Barthels and Das, 2020). The review of the evidence in previous literature suggests that the primary subtype of stroke is ischemic stroke, aligning with epidemiological trends (GBD Parkinson’s Disease Collaborators, 2016). Additionally, MRI studies of acupuncture for stroke address multiple post-stroke dysfunctions, such as motor, speech, cognitive, swallowing, and sensory impairments, with a significant emphasis on motor impairments. Our results similarly support this conclusion. Post-stroke motor impairment is a common complication, with over 70% of stroke survivors experiencing motor dysfunctions (Wang et al., 2017). These studies make the functional abnormalities and reorganization between brain regions or networks observable.

### 4.2 Trial design

Several commonly used experimental design methods are described as follows: (1) Single-group, single-session design: A group of patients with stroke participated in the study and received a single intervention and single-signal acquisition to observe immediate brain activity. (2) Single-group pre-post control design: A group of patients with stroke participated in the study and received a period of intervention, with signal acquisition conducted before and after the intervention to observe brain activity over time. (3) Two-group single-session design: Two groups of patients with stroke participated in the study, each receiving different interventions and undergoing single-signal acquisition after randomization, allowing the observation of immediate brain activity. (4) Two-group pre-post control design: Two groups of patients with stroke participated in the study, with different interventions administered after randomization. Signal-acquisition was performed before and after the intervention to observe brain activity over time. Healthy controls could/could not be included in this study.

Additionally, two other studies focused on natural recovery state design (Zhao et al., 2017; Stinear et al., 2020). These studies compared brain activity in patients with stroke under natural recovery conditions and observed brain activity changes over time. During this period, no acupuncture intervention was performed, although the acupuncture BLOCK intervention was used to stimulate brain activity at each visiting viewpoint. Five studies employed two-group cross-over control designs (Chang et al., 2003; Zhang et al., 2014; Chen et al., 2015; Liao et al., 2016; Chen C. et al., 2024; Chen T. et al., 2024), which involved two groups of single-session designs with another intervention after the washout period to create two groups of cross-control studies. Consequently, immediate brain activity was observed. This design was mostly used in the group design of acupuncture and SA acupuncture controls. Two studies used a single-group, multi-control design (Si et al., 2013; Chen et al., 2014), where a group of patients with stroke received several different interventions in sequence, separated by a washout period. In the two-group crossover control and single-group multi-control designs, each patient received two different interventions, reducing the sample size and eliminating individual differences.

### 4.3 Magnetic resonance imaging intervention design

Resting-state (rs)-fMRI and task-state fMRI are the two primary paradigms for fMRI studies.

1. Resting-state mode: This mode refers to the spontaneous regulation activity of neurones in the specific area of the brain observed using MRI when subjects are awake and at rest, without specific brain activity. This state exhibits a significant degree of stability. Rs-MRI is used to investigate changes in fMRI signals before and after acupuncture treatment. This approach is suitable for single-group pre-post control and two-group pre-post control designs to explore how acupuncture treatment affects brain activity over time.
2. Task-state mode: Acupuncture is used as a passive input stimulus to investigate alterations in brain activation during and after needle stimulation. This model investigates both the immediate and sustained effects of acupuncture, necessitating a series of predetermined acupuncture or non-acupuncture task stimuli to induce changes in the brain regions. ① Classical BLOCK paradigm: Each experimental session alternates between resting-state and stimulation blocks, including acupuncture (Huang et al., 2013) and non-acupuncture task BLOCKs (like finger movement, language test) (Xu et al., 2022). Acupuncture BLOCK applies to single-group single-session design, two-group single-session design, two-group crossover control design, single-group multi-control design, and natural recovery state design. It explores altered brain activity as an immediate effect of acupuncture treatment. Conversely, non-acupuncture task BLOCK applies to the single-group pre-post control design and two-group pre-post control design, exploring altered brain activity during the task after a period of acupuncture treatment. ② NRER paradigm: Initially, an acupuncture needle is inserted at the acupoint, rested for 1 min, manipulated for 1 min, and subsequently left inserted for another 8 min. This approach is applicable to the Single-group single-session design and two groups single-session design to investigate sustained effects after instant acupuncture administration (Xiao et al., 2020). Therefore, we employed the BLOCK design to avoid the cumulative and confounding effects of acupuncture. ③ MIX-BLOCK paradigm: A series of non-acupuncture tasks are combined with acupuncture, often stimulating specific brain activities through body movements during acupuncture. For example, researchers have explored the differences in brain activity in patients with stroke during fist-grasping tasks with non-acupuncture, acupuncture, and SA (Lu et al., 2023). It is mostly applicable to a single-group, single-session design, making brain activity more complex when receiving a single stimulus and potentially confusing the exact acupuncture effect. ④ Pre-post paradigm: The MRI signal scan was not performed during the acupuncture intervention; however, before and after the acupuncture intervention, the subject was required to remain still or perform a task, with signal changes attributed to the acupuncture event. It is mostly applicable to single-group, single-session and two-group single-session designs. Several studies have conducted swallowing BLOCK scans before and after acupuncture, revealing increased brain function activation areas post-acupuncture compared with pre-acupuncture. These findings suggest that acupuncture at the tongue root can enhance the involvement of additional brain regions during swallowing (Schockert et al., 2010; Liu et al., 2019). ⑤ Single-Block paradigm: The resting state and acupuncture stimulation events are only repeated once, which avoids the signal baseline elevation caused by cumulative acupuncture effects in the BLOCK design, reducing the accumulation of acupuncture effect to some extent.

Our results showed that the most frequently observed patterns were the resting-state, classical BLOCK, non-repeated event-related (NRER), and MIX-BLOCK paradigms. Each research paradigm has different objectives and purposes.

### 4.4 Control design

Our findings indicate that the control design of acupuncture in stroke MRI studies primarily addresses two major research objectives: the mechanism of acupuncture and its efficacy. The investigation of mechanisms includes comparisons such as VA / VA combined with other therapies, VA versus HC, VA versus SA, self-control before and after, and VA 1 versus VA 2. The purpose of SA is to prove that the positive effects of acupuncture treatment are due to the placebo effect. Of the included studies, 18 used the following three types of SA: (1) acupuncture treatment at non-acupoints, with two studies emphasizing that non-meridian points should be avoided De-qi (Zhang J. et al., 2023; Zhang J. S. et al., 2023; Zhang Y. et al., 2023; Chen C. et al., 2024; Chen T. et al., 2024); (2) mild stimulation such as superficial acupuncture (Chen et al., 2014; Wang et al., 2023); and (3) tactile stimulation (Chen et al., 2015), use of non-penetrating and flexible needles (Huang et al., 2013), and application of no-current electric stimulators (Schaechter et al., 2007). SA plays a critical role in understanding and analysing the effects of acupuncture; however, the overall quality of reporting is suboptimal. SA reports are recommended based on the guidelines outlined in the fake needle report list (Jiang et al., 2017). The purpose of the healthy population is to demonstrate the functional specificity of acupuncture, suggesting that acupuncture exerts a significant effect under pathological conditions. The therapeutic efficacy of acupuncture is attained by restoring the internal environmental balance. Imaging studies conducted in healthy subjects may reflect pre-intervention brain homeostasis in patients, thereby providing a more comprehensive understanding of the mechanisms underlying the efficacy of acupuncture.

Several potential factors were considered regarding efficacy, including needle timing, needle retention time, Deqi and non-Deqi, and different acupoints. A study investigating the differential activation responses of the brains of patients with stroke in the morning and afternoon revealed a stronger activation effect in the morning than in the afternoon (Ma et al., 2023). Another study categorized participants into groups based on needle retention times of 1, 2, and 3 min, utilizing a consistent acupoint prescription across groups. These findings indicate that varying the retention time of acupuncture treatment results in distinct neural effects in patients with stroke, with the volume of the activated voxel cluster showing a positive correlation with the duration of acupuncture (Gao et al., 2015). Several studies have demonstrated that the Deqi group exhibits significant activation in relevant brain regions compared with the non-Deqi group (Zhou et al., 2023). Additionally, researchers have compared the effects of cardiac and pericardial meridian acupoints on disease and brain mechanisms, with results showing a better effect of pericardial meridian acupoints than that of cardiac meridian acupoints (Li et al., 2015). Furthermore, the central effect of the Baihui acupoint is more potent than that of Yanglingquan in ameliorating memory impairment post-stroke, potentially due to the enhanced functional connectivity of the hippocampus and the brain network between the frontal and parietal lobes (Zhang et al., 2019). These studies provide neuroimaging evidence supporting the underlying mechanisms in acupuncture.

### 4.5 Outcome indicators

MRI is an emerging neuroimaging method for exploring the central mechanism of acupuncture in patients with stroke. Our review indicates that multimodal MRI technologies are increasingly attracting research attention.

In neuroimaging, fMRI is widely used to explore brain function, behavior, perception, and emotions. Our findings indicate that these trends are significantly expanding both transversely and longitudinally. Researchers’ observation index has broadened from initially focusing on local neural activity within specific brain regions to including the effects on different regions within the ipsilateral and contralateral hemispheres, extending to the overall brain network. Magnetic resonance spectroscopy imaging (MRS/1H-MRS) is primarily employed to quantify the concentration of specific metabolites in the brain or other tissues and is frequently used to investigate the metabolic state and pathological lesions of the brain. Different lesion areas can be selected for detection based on stroke complications. Additionally, dynamic, non-invasive monitoring of critical brain metabolites can be conducted, thereby providing robust evidence supporting molecular imaging in the acupuncture treatment of stroke. Diffusion magnetic resonance imaging (DWI/DTI) is predominantly used to delineate the structural architecture and white matter pathways within the brain and investigate acupuncture as a treatment modality for motor dysfunction following stroke. This imaging technique enables quantitative analysis of nerve fiber damage and motor dysfunction, which is crucial for understanding acupunctures’ efficacy in motor function rehabilitation. DTI is often combined with fMRI to explore the interrelationships between the brain structure and function. Magnetic resonance perfusion imaging, including arterial spin labelling (ASL) and perfusion-weighted imaging (PWI), offers a rapid assessment of haemodynamic alterations in the brain tissue. ASL perfusion imaging is a common technique for measuring cerebral blood flow (CBF). In patients with stroke, variations in infarct location correspond to differences in cerebral blood flow signals. Alterations in local cerebral blood flow within specific brain regions may constitute a critical neural mechanism underlying functional impairment. Additionally, acupuncture may facilitate neural repair by augmenting the local cerebral blood flow. Structural magnetic resonance imaging (sMRI) provides high-resolution images of the brain and other anatomical structures, facilitating the detailed observation of anatomical features. This modality is extensively utilized for diagnostic purposes, disease monitoring, and investigating structural changes in the brain.

Thus, fMRI focuses on brain activity and function, MRS/1H-MRS on chemical and metabolic states, DWI/DTI on white matter structure and function, sMRI on anatomical structures, and ASL/PWI on cerebral blood flow. Collectively, these neuroimaging techniques provide robust scientific and quantitative evidence supporting the efficacy of acupuncture in stroke treatment by addressing various dimensions, such as functional activity, metabolic processes, structural integrity, and cerebral perfusion. This multifaceted approach highlights the comprehensive therapeutic potential of acupuncture for stroke rehabilitation.

This review presents the first systematic review using evidence map to illustrate the current state of neuroimaging research on the acupuncture treatment of stroke. It provides a significant reference for future scholarly research, offering imaging evidence that elucidates the brain network mechanism of acupuncture interventions in stroke. However, the current study has certain limitations, including a predominance of single-center studies with small sample sizes and a lack of long-term follow-up. Future research should consider the implementation of multicenter, large-sample, clinical randomized controlled studies to further investigate the clinical efficacy of acupuncture and the potential brain network mechanisms that could enhance stroke recovery.
