# Supplementary material for: Overview of evidence-based research on acupuncture for stroke treatment using magnetic resonance imaging technology
Source: Front Neurosci. 2024 Nov 25;18:1495435. doi: 10.3389/fnins.2024.1495435 (PMC11625797; doi:10.3389/fnins.2024.1495435)

**Supplementary File 1. Search strategies of each database.**

**PubMed**

#1 (Acupuncture OR Acupuncture Therapy OR Moxibustion[MeSH Terms])

#2 (Acupuncture[Title/Abstract] OR Acupuncture Therapy[Title/Abstract] OR Acupuncture Treatment[Title/Abstract] OR Treatment, Acupuncture[Title/Abstract] OR Therapy, Acupuncture[Title/Abstract] OR Manual Acupuncture[Title/Abstract] OR Acupuncture-moxibustion[Title/Abstract] OR Moxibustion[Title/Abstract] OR Moxabustion[Title/Abstract] OR Warm needle[Title/Abstract] OR Warm Acupuncture[Title/Abstract] OR Thermoacupuncture[Title/Abstract] OR Electroacupuncture[Title/Abstract] OR Electro-acupuncture[Title/Abstract] OR electric acupuncture[Title/Abstract] OR Acupuncture Point[Title/Abstract] OR Point, Acupuncture[Title/Abstract] OR Acupoint[Title/Abstract] OR Pharmacopuncture[Title/Abstract] OR Pharmacoacupuncture Treatment[Title/Abstract] OR Treatment, Pharmacoacupuncture[Title/Abstract] OR Pharmacoacupuncture Therapy[Title/Abstract] OR Therapy, Pharmacoacupuncture[Title/Abstract] OR Acupoint injection[Title/Abstract] OR acupuncture injection[Title/Abstract] OR pharmaco-acupuncture[Title/Abstract] OR auricular needle[Title/Abstract] OR Acupunctures, Ear[Title/Abstract] OR Ear Acupuncture[Title/Abstract] OR Ear needle[Title/Abstract] OR earlobe acupuncture[Title/Abstract] OR Auricular Acupuncture[Title/Abstract] OR Auriculotherapy[Title/Abstract] OR Acupuncture, Auricular[Title/Abstract] OR auricular plaster therapy[Title/Abstract] OR auricular point sticking[Title/Abstract] OR Auricular pressure[Title/Abstract] OR Fire needle[Title/Abstract] OR Fire acupuncture[Title/Abstract] OR Acupoint catgut embedding[Title/Abstract] OR Scalp acupuncture[Title/Abstract] OR Scalp needle[Title/Abstract] OR Scalp electroacupuncture[Title/Abstract] OR scalp stimulation[Title/Abstract] OR Eye needle[Title/Abstract] OR Eye acupuncture[Title/Abstract] OR Abdominal acupuncture[Title/Abstract] OR Abdominal needle[Title/Abstract] OR filiform needle[Title/Abstract] OR silver needle[Title/Abstract] OR three-edged needle[Title/Abstract] OR intradermal needle[Title/Abstract] OR Point application[Title/Abstract] OR needle-embedding[Title/Abstract] OR Catgut Embedding[Title/Abstract] OR pricking therapy[Title/Abstract] OR point injection[Title/Abstract] OR Skin Acupuncture[Title/Abstract] OR transcutanclus electrical acupoint stimulation[Title/Abstract] OR TEAS[Title/Abstract] OR electrical acupoint stimulation[Title/Abstract] OR Acupuncture Point[Title/Abstract] OR Acupoint[Title/Abstract])

#3 #1 OR #2

#4 (Stroke OR Brain Infarction OR Brain Ischemia OR Cerebral Infarction OR Cerebral Hemorrhage OR Apoplexy OR Intracerebral Hemorrhage[MeSH Terms])

#5 (Stroke*[Title/Abstract] OR Apoplexy[Title/Abstract] OR Cerebrovascular Apoplexy[Title/Abstract] OR Apoplexy, Cerebrovascular[Title/Abstract] OR Cerebrovascular Stroke*[Title/Abstract] OR Cerebral Stroke*[Title/Abstract] OR Acute Stroke*[Title/Abstract] OR Embolic Stroke*[Title/Abstract] OR Wake up Stroke*[Title/Abstract] OR Ischemic Stroke*[Title/Abstract] OR Thrombotic Stroke*[Title/Abstract] OR Brain Ischemia[Title/Abstract] OR Stroke*, Cerebrovascular[Title/Abstract] OR Stroke*, Cerebral[Title/Abstract] OR Stroke*, Acute[Title/Abstract] OR Brain Infarction[Title/Abstract] OR Cerebral Infarct*[Title/Abstract] OR Intracerebral Hemorrhage*[Title/Abstract] OR Cerebral Hemorrhage*[Title/Abstract] OR Cerebral Brain Hemorrhage*[Title/Abstract] OR Hemorrhagic stroke*[Title/Abstract] OR Hypertensive Intracerebral Hemorrhage*[Title/Abstract] OR Hemorrhage*, Cerebrum[Title/Abstract] OR Cerebrum Hemorrhage*[Title/Abstract] OR Cerebral Parenchymal Hemorrhage*[Title/Abstract] OR Hemorrhage*, Cerebral Parenchymal[Title/Abstract] OR Parenchymal Hemorrhage*, Cerebral[Title/Abstract] OR Hemorrhage*, Intracerebral[Title/Abstract] OR Hemorrhage*, Cerebral[Title/Abstract] OR Cerebral Hemorrhage*[Title/Abstract] OR Brain Hemorrhage*, Cerebral[Title/Abstract] OR Hemorrhage*, Cerebral Brain[Title/Abstract] OR Cerebrovascular Accident*[Title/Abstract] OR Acute Cerebrovascular Accident*[Title/Abstract] OR CVA*[Title/Abstract] OR Vascular Accident*, Brain[Title/Abstract] OR Brain Vascular Accident*[Title/Abstract])

#6 #4 OR #5

#7 MRI[Title/Abstract] OR magnetic resonance imaging[Title/Abstract] OR fMRI[Title/Abstract] OR functional MRI*[Title/Abstract] OR functional magnetic resonance imaging[Title/Abstract] OR neuroimaging[Title/Abstract] OR MRI*, Functional[Title/Abstract] OR Magnetic Resonance Imaging, Functional[Title/Abstract] OR BOLD[Title/Abstract] OR blood oxygen level dependent[Title/Abstract] OR ReHo[Title/Abstract] OR regional homogeneity[Title/Abstract] OR the amplitude of low-frequency fluctuation[Title/Abstract] OR ALFF[Title/Abstract] OR fALFF[Title/Abstract] OR fractional ALFF[Title/Abstract] OR fractional amplitude of low-frequency[Title/Abstract] OR white matter[Title/Abstract] OR voxel-based analysis[Title/Abstract] OR VBM[Title/Abstract] OR voxel-based morphometry[Title/Abstract] OR Freesurfer[Title/Abstract] OR surface-based morphometry[Title/Abstract] OR cortical thickness[Title/Abstract] OR surface area[Title/Abstract] OR cortical volume[Title/Abstract] OR gray matter volume[Title/Abstract] OR gray matter density[Title/Abstract] OR DTI[Title/Abstract] OR Difusion tensor imaging[Title/Abstract] OR Region of interest[Title/Abstract] OR ROI[Title/Abstract] OR Independent component analysis[Title/Abstract] OR ICA[Title/Abstract] OR Functional connectivity[Title/Abstract] OR FC[Title/Abstract] OR Arterial spin labeling[Title/Abstract] OR ASL[Title/Abstract]

#8 #6 AND #3 AND #7

**results：n=178**


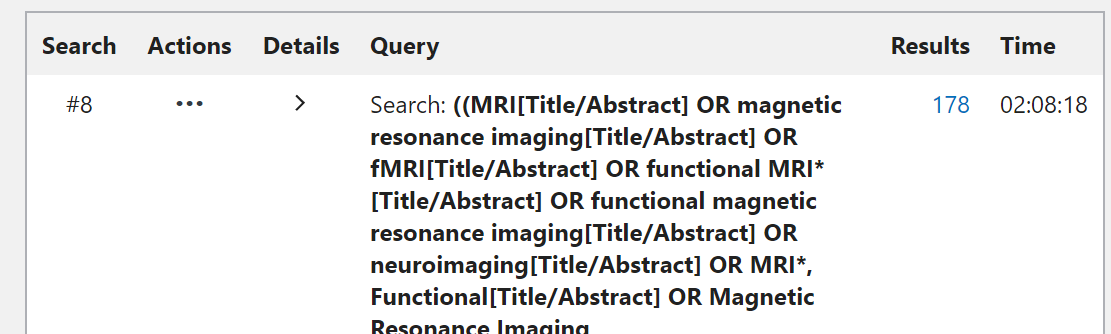


**Embase**

#1 'acupuncture'/exp OR 'moxibustion'/exp OR 'acupuncture therapy'/exp OR acupuncture:ti,ab,kw OR 'acupuncture therapy':ti,ab,kw OR 'acupuncture treatment':ti,ab,kw OR 'treatment, acupuncture':ti,ab,kw OR 'therapy, acupuncture':ti,ab,kw OR 'manual acupuncture':ti,ab,kw OR 'acupuncture moxibustion':ti,ab,kw OR moxibustion:ti,ab,kw OR moxabustion:ti,ab,kw OR 'warm needle':ti,ab,kw OR 'warm acupuncture':ti,ab,kw OR thermoacupuncture:ti,ab,kw OR electroacupuncture:ti,ab,kw OR 'electro acupuncture':ti,ab,kw OR 'electric acupuncture':ti,ab,kw OR 'point, acupuncture':ti,ab,kw OR pharmacopuncture:ti,ab,kw OR 'pharmacoacupuncture treatment':ti,ab,kw OR 'treatment, pharmacoacupuncture':ti,ab,kw OR 'pharmacoacupuncture therapy':ti,ab,kw OR 'therapy, pharmacoacupuncture':ti,ab,kw OR 'acupoint injection':ti,ab,kw OR 'acupuncture injection':ti,ab,kw OR 'pharmaco acupuncture':ti,ab,kw OR 'auricular needle':ti,ab,kw OR 'acupunctures, ear':ti,ab,kw OR 'ear acupuncture':ti,ab,kw OR 'ear needle':ti,ab,kw OR 'earlobe acupuncture':ti,ab,kw OR 'auricular acupuncture':ti,ab,kw OR auriculotherapy:ti,ab,kw OR 'acupuncture, auricular':ti,ab,kw OR 'auricular plaster therapy':ti,ab,kw OR 'auricular point sticking':ti,ab,kw OR 'auricular pressure':ti,ab,kw OR 'fire needle':ti,ab,kw OR 'fire acupuncture':ti,ab,kw OR 'acupoint catgut embedding':ti,ab,kw OR 'scalp acupuncture':ti,ab,kw OR 'scalp needle':ti,ab,kw OR 'scalp electroacupuncture':ti,ab,kw OR 'scalp stimulation':ti,ab,kw OR 'eye needle':ti,ab,kw OR 'eye acupuncture':ti,ab,kw OR 'abdominal acupuncture':ti,ab,kw OR 'abdominal needle':ti,ab,kw OR 'filiform needle':ti,ab,kw OR 'silver needle':ti,ab,kw OR 'three-edged needle':ti,ab,kw OR 'intradermal needle':ti,ab,kw OR 'point application':ti,ab,kw OR 'needle embedding':ti,ab,kw OR 'catgut embedding':ti,ab,kw OR 'pricking therapy':ti,ab,kw OR 'point injection':ti,ab,kw OR 'skin acupuncture':ti,ab,kw OR 'transcutanclus electrical acupoint stimulation':ti,ab,kw OR teas:ti,ab,kw OR 'electrical acupoint stimulation':ti,ab,kw OR 'acupuncture point':ti,ab,kw OR acupoint:ti,ab,kw

#2 'stroke'/exp OR 'brain infarction'/exp OR 'brain ischemia'/exp OR 'cerebral infarction'/exp OR 'cerebral hemorrhage'/exp OR 'apoplexy'/exp OR 'intracerebral hemorrhage'/exp OR stroke*:ti,ab,kw OR apoplexy:ti,ab,kw OR 'cerebrovascular apoplexy':ti,ab,kw OR 'apoplexy, cerebrovascular':ti,ab,kw OR 'cerebrovascular stroke*':ti,ab,kw OR 'cerebral stroke*':ti,ab,kw OR 'acute stroke*':ti,ab,kw OR 'embolic stroke*':ti,ab,kw OR 'wake up stroke*':ti,ab,kw OR 'ischemic stroke*':ti,ab,kw OR 'thrombotic stroke*':ti,ab,kw OR 'brain ischemia':ti,ab,kw OR 'stroke*, cerebrovascular':ti,ab,kw OR 'stroke*, cerebral':ti,ab,kw OR 'stroke*, acute':ti,ab,kw OR 'brain infarction':ti,ab,kw OR 'cerebral infarct*':ti,ab,kw OR 'intracerebral hemorrhage*':ti,ab,kw OR 'cerebral brain hemorrhage*':ti,ab,kw OR 'hemorrhagic stroke*':ti,ab,kw OR 'hypertensive intracerebral hemorrhage*':ti,ab,kw OR 'hemorrhage*, cerebrum':ti,ab,kw OR 'cerebrum hemorrhage*':ti,ab,kw OR 'cerebral parenchymal hemorrhage*':ti,ab,kw OR 'hemorrhage*, cerebral parenchymal':ti,ab,kw OR 'parenchymal hemorrhage*, cerebral':ti,ab,kw OR 'hemorrhage*, intracerebral':ti,ab,kw OR 'hemorrhage*, cerebral':ti,ab,kw OR 'cerebral hemorrhage*':ti,ab,kw OR 'brain hemorrhage*, cerebral':ti,ab,kw OR 'hemorrhage*, cerebral brain':ti,ab,kw OR 'cerebrovascular accident*':ti,ab,kw OR 'acute cerebrovascular accident*':ti,ab,kw OR cva*:ti,ab,kw OR 'vascular accident*, brain':ti,ab,kw OR 'brain vascular accident*':ti,ab,kw

#3 mri:ti,ab,kw OR 'magnetic resonance imaging':ti,ab,kw OR fmri:ti,ab,kw OR 'functional mri*':ti,ab,kw OR 'functional magnetic resonance imaging':ti,ab,kw OR neuroimaging:ti,ab,kw OR 'mri*, functional':ti,ab,kw OR 'magnetic resonance imaging, functional':ti,ab,kw OR bold:ti,ab,kw OR 'blood oxygen level dependent':ti,ab,kw OR reho:ti,ab,kw OR 'regional homogeneity':ti,ab,kw OR 'the amplitude of low-frequency fluctuation':ti,ab,kw OR alff:ti,ab,kw OR falff:ti,ab,kw OR 'fractional alff':ti,ab,kw OR 'fractional amplitude of low-frequency':ti,ab,kw OR 'white matter':ti,ab,kw OR 'voxel-based analysis':ti,ab,kw OR vbm:ti,ab,kw OR 'voxel-based morphometry':ti,ab,kw OR freesurfer:ti,ab,kw OR 'surface-based morphometry':ti,ab,kw OR 'cortical thickness':ti,ab,kw OR 'surface area':ti,ab,kw OR 'cortical volume':ti,ab,kw OR 'gray matter volume':ti,ab,kw OR 'gray matter density':ti,ab,kw OR dti:ti,ab,kw OR 'difusion tensor imaging':ti,ab,kw OR 'region of interest':ti,ab,kw OR roi:ti,ab,kw OR 'independent component analysis':ti,ab,kw OR ica:ti,ab,kw OR 'functional connectivity':ti,ab,kw OR fc:ti,ab,kw OR 'arterial spin labeling':ti,ab,kw OR asl:ti,ab,kw

#4 #1 AND #2 AND #3

**results：n=216**


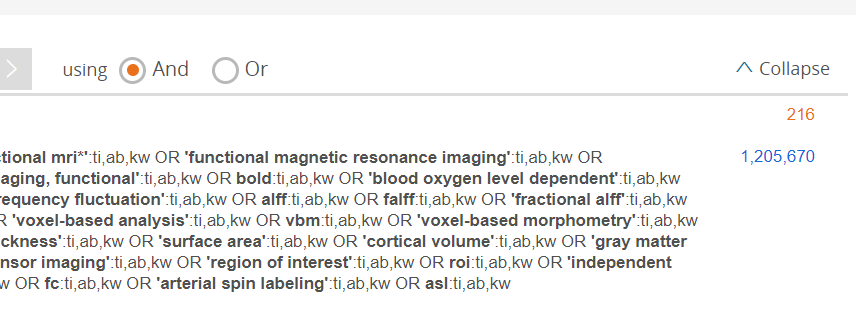


**Cochrane**

#1 MeSH descriptor: [Acupuncture] explode all trees

#2 MeSH descriptor: [Acupuncture Therapy] explode all trees

#3 MeSH descriptor: [Moxibustion] explode all trees

#4(Acupuncture OR Acupuncture Therapy OR Acupuncture Treatment OR Treatment, Acupuncture OR Therapy, Acupuncture OR Manual Acupuncture OR Acupuncture-moxibustion OR Moxibustion OR Moxabustion OR Warm needle OR Warm Acupuncture OR Thermoacupuncture OR Electroacupuncture OR Electro-acupuncture OR electric acupuncture OR Acupuncture Point OR Point, Acupuncture OR Acupoint OR Pharmacopuncture OR Pharmacoacupuncture Treatment OR Treatment, Pharmacoacupuncture OR Pharmacoacupuncture Therapy OR Therapy, Pharmacoacupuncture OR Acupoint injection OR acupuncture injection OR pharmaco-acupuncture OR auricular needle OR Acupunctures, Ear OR Ear Acupuncture OR Ear needle OR earlobe acupuncture OR Auricular Acupuncture OR Auriculotherapy OR Acupuncture, Auricular OR auricular plaster therapy OR auricular point sticking OR Auricular pressure OR Fire needle OR Fire acupuncture OR Acupoint catgut embedding OR Scalp acupuncture OR Scalp needle OR Scalp electroacupuncture OR scalp stimulation OR Eye needle OR Eye acupuncture OR Abdominal acupuncture OR Abdominal needle OR filiform needle OR silver needle OR three-edged needle OR intradermal needle OR Point application OR needle-embedding OR Catgut Embedding OR pricking therapy OR point injection OR Skin Acupuncture OR transcutanclus electrical acupoint stimulation OR TEAS OR electrical acupoint stimulation OR Acupuncture Point OR Acupoint):ti,ab,kw

#5 #1 OR #2 OR #3 OR #4

#6 MeSH descriptor: [Stroke] explode all trees

#7 MeSH descriptor: [Cerebral Hemorrhage] explode all trees

#8 MeSH descriptor: [Brain Infarction] explode all trees

#9 MeSH descriptor: [Cerebral Infarction] explode all trees

#10 MeSH descriptor: [Brain Ischemia] explode all trees

#11 (Stroke* OR Apoplexy OR Cerebrovascular Apoplexy OR Apoplexy, Cerebrovascular OR Cerebrovascular Stroke* OR Cerebral Stroke* OR Acute Stroke* OR Embolic Stroke* OR Wake up Stroke* OR Ischemic Stroke* OR Thrombotic Stroke* OR Brain Ischemia OR Stroke*, Cerebrovascular OR Stroke*, Cerebral OR Stroke*, Acute OR Brain Infarction OR Cerebral Infarct* OR Intracerebral Hemorrhage* OR Cerebral Hemorrhage* OR Cerebral Brain Hemorrhage* OR Hemorrhagic stroke* OR Hypertensive Intracerebral Hemorrhage* OR Hemorrhage*, Cerebrum OR Cerebrum Hemorrhage* OR Cerebral Parenchymal Hemorrhage* OR Hemorrhage*, Cerebral Parenchymal OR Parenchymal Hemorrhage*, Cerebral OR Hemorrhage*, Intracerebral OR Hemorrhage*, Cerebral OR Cerebral Hemorrhage* OR Brain Hemorrhage*, Cerebral OR Hemorrhage*, Cerebral Brain OR Cerebrovascular Accident* OR Acute Cerebrovascular Accident* OR CVA* OR Vascular Accident*, Brain OR Brain Vascular Accident*):ti,ab,kw

#12 #6 OR #7 OR #8 OR #9 OR #10 OR #11

#13 (MRI OR magnetic resonance imaging OR fMRI OR functional MRI* OR functional magnetic resonance imaging OR neuroimaging OR MRI*, Functional OR Magnetic Resonance Imaging, Functional OR BOLD OR blood oxygen level dependent OR ReHo OR regional homogeneity OR the amplitude of low-frequency fluctuation OR ALFF OR fALFF OR fractional ALFF OR fractional amplitude of low-frequency OR white matter OR voxel-based analysis OR VBM OR voxel-based morphometry OR Freesurfer OR surface-based morphometry OR cortical thickness OR surface area OR cortical volume OR gray matter volume OR gray matter density OR DTI OR Difusion tensor imaging OR Region of interest OR ROI OR Independent component analysis OR ICA OR Functional connectivity OR FC OR Arterial spin labeling OR ASL):ti,ab,kw

#14 #5 AND #12 AND #13

**results：n=470**


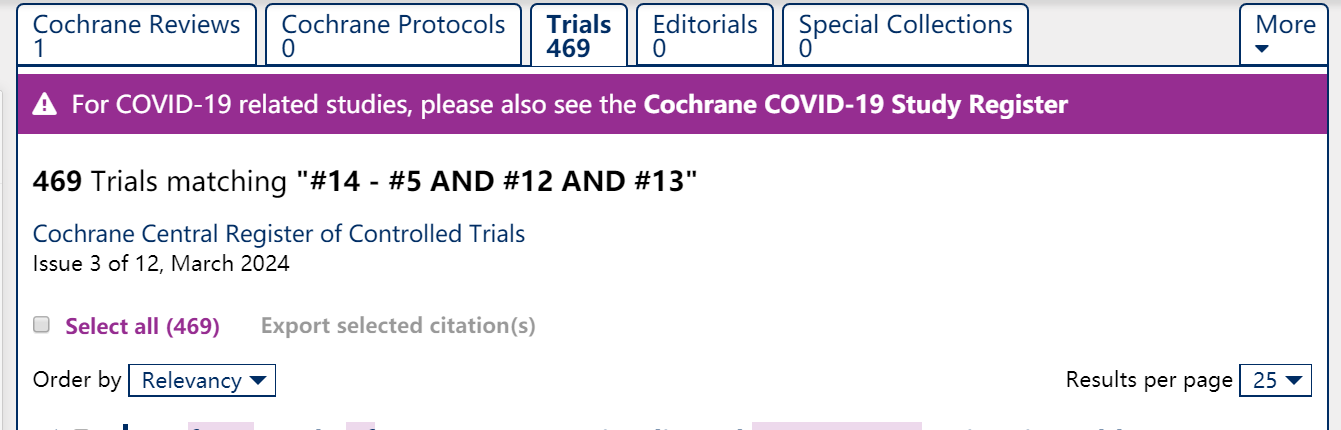


**CNKI**

(SU%=('针刺'+'电针'+'针灸'+'刺法'+'经皮穴位电刺激'+'隔姜灸'+'隔药灸'+'隔附子饼灸'+'体针'+'耳针'+'头针'+'毫针'+'隔盐灸'+'耳穴贴压'+'热敏灸'+'梅花针'+'穴位敷贴'+'穴位贴敷'+'天灸'+'针刺治疗'+'针灸疗法'+'穴位埋线'+'火针'+'穴位注射'+'艾灸'+'灸法'+'灸疗'+'灸术'+'温针'+'针法'+'三棱针'+'皮肤针'+'芒针'+'眼针'+'手针'+'足针'+'腕踝针'+'平衡针'+'揿针'+'皮内针'+'腹针'+'舌针'+'项针')) AND (SU%=('中风' +'卒中'+'脑梗死'+'脑梗塞'+'脑出血')) AND (SU%=('磁共振' +'影像'+'fMRI' + 'MRI' +'灰质'+'白质'+'体素' +'独立成分分析'+'ICA' +'ROI'+'种子相关分析' +'ALFF'+'低频振幅'+'fALFF'+'ReHo'+'局域一致性'+'血氧水平依赖性' +'BOLD'+'基于体素的分析' +'基于体素的形态测量'+'皮质厚度'))

**results：n=230**

**
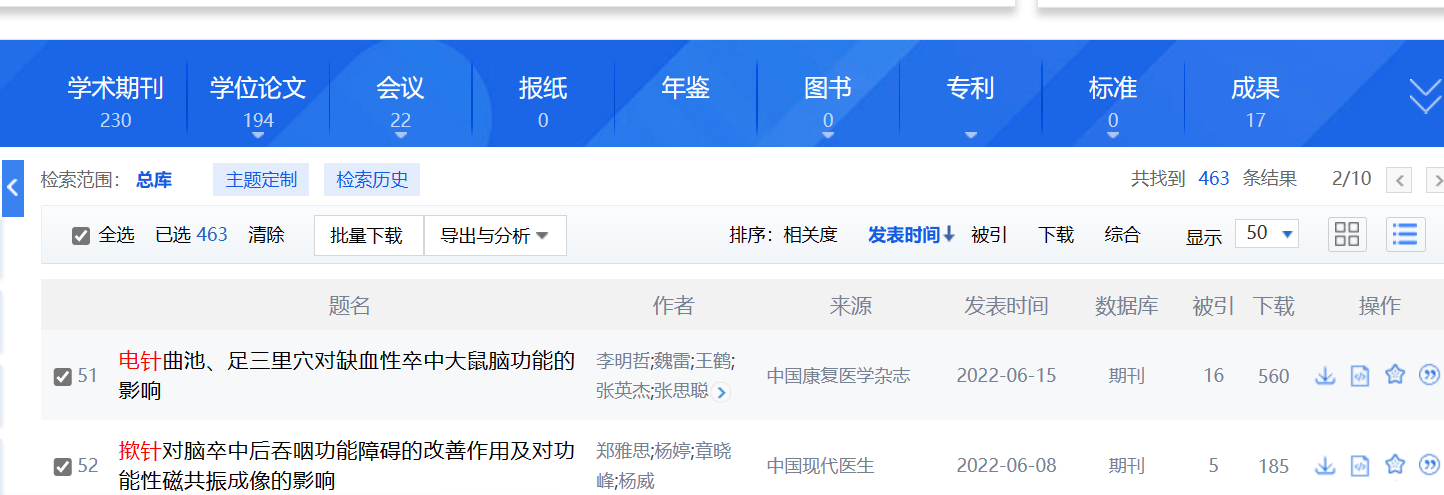
**

**VIP**

(M=(针刺 OR 电针 OR 针灸 OR 刺法 OR 经皮穴位电刺激 OR 隔姜灸 OR 隔药灸 OR 隔附子饼灸 OR 体针 OR 耳针 OR 头针 OR 毫针 OR 隔盐灸 OR 耳穴贴压 OR 热敏灸 OR 梅花针 OR 穴位敷贴 OR 穴位贴敷 OR 天灸 OR 针刺治疗 OR 针灸疗法 OR 穴位埋线 OR 火针 OR 穴位注射 OR 艾灸 OR 灸法 OR 灸疗 OR 灸术 OR 温针 OR 针法 OR 三棱针 OR 皮肤针 OR 芒针 OR 眼针 OR 手针 OR 足针 OR 腕踝针 OR 平衡针 OR 揿针 OR 皮内针 OR 腹针 OR 舌针 OR 项针)) AND (M=(中风 OR 卒中 OR 脑梗死 OR 脑梗塞 OR 脑出血)) AND (M=(磁共振 OR 影像 OR fMRI OR MRI OR 灰质 OR 白质 OR 体素 OR 独立成分分析 OR ICA OR ROI OR 种子相关分析 OR ALFF OR 低频振幅 OR fALFF OR ReHo OR 局域一致性 OR 血氧水平依赖性 OR BOLD OR 基于体素的分析 OR 基于体素的形态测量 OR 皮质厚度))

**results：n=396**


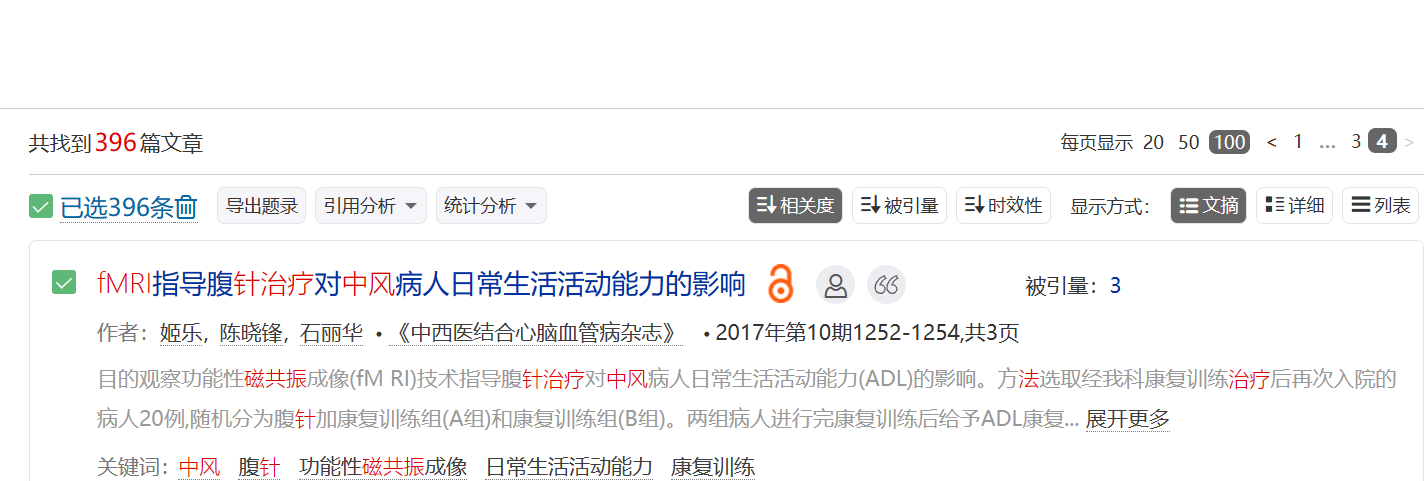


**万方**

主题:(“针刺” OR “电针” OR “针灸” OR “刺法” OR “经皮穴位电刺激” OR “隔姜灸” OR “隔药灸” OR “隔附子饼灸” OR “体针” OR “耳针” OR “头针” OR “毫针” OR “隔盐灸” OR “耳穴贴压” OR “热敏灸” OR “梅花针” OR “穴位敷贴” OR “穴位贴敷” OR “天灸” OR “针刺治疗” OR “针灸疗法” OR “穴位埋线” OR “火针” OR “穴位注射” OR “艾灸” OR “灸法” OR “灸疗” OR “灸术” OR “温针” OR “针法” OR “三棱针” OR “皮肤针” OR “芒针” OR “眼针” OR “手针” OR “足针” OR “腕踝针” OR “平衡针” OR “揿针” OR “皮内针” OR “腹针” OR “舌针” OR “项针”) AND 主题:(“中风” OR “卒中” OR “脑梗死” OR “脑梗塞” OR “脑出血”) AND 主题:(“磁共振” OR “影像” OR “fMRI” OR “MRI” OR “灰质” OR “白质” OR “体素” OR “独立成分分析” OR “ICA” OR “ROI” OR “种子相关分析” OR “ALFF” OR “低频振幅” OR “fALFF” OR “ReHo” OR “局域一致性” OR “血氧水平依赖性” OR “BOLD” OR “基于体素的分析” OR “基于体素的形态测量” OR “皮质厚度”)

**results：n=628**


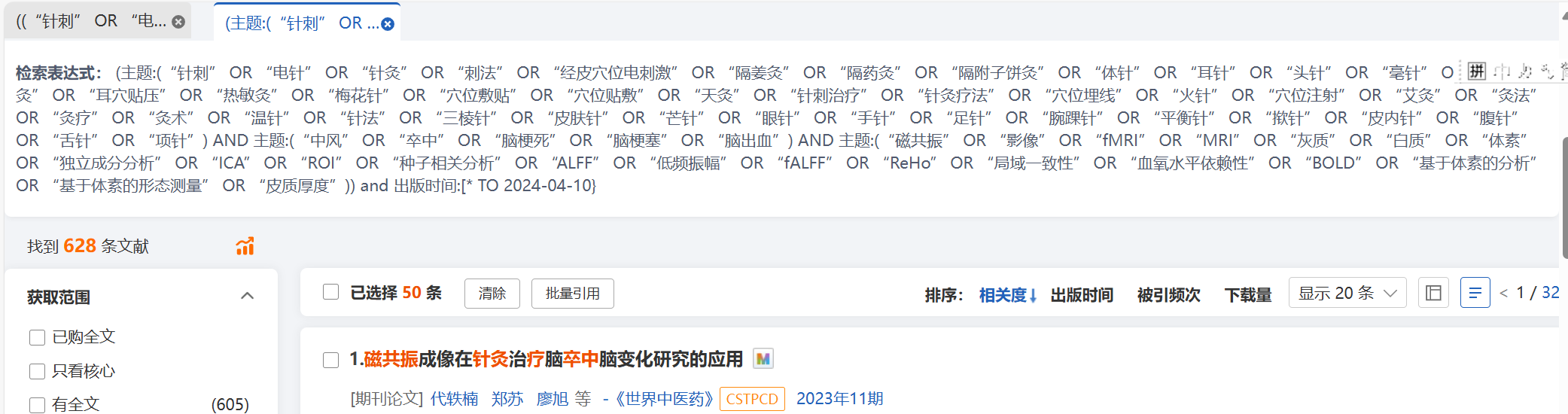


**CBM**

(针刺 OR 电针 OR 针灸 OR 刺法 OR 经皮穴位电刺激 OR 隔姜灸 OR 隔药灸 OR 隔附子饼灸 OR 体针 OR 耳针 OR 头针 OR 毫针 OR 隔盐灸 OR 耳穴贴压 OR 热敏灸 OR 梅花针 OR 穴位敷贴 OR 穴位贴敷 OR 天灸 OR 针刺治疗 OR 针灸疗法 OR 穴位埋线 OR 火针 OR 穴位注射 OR 艾灸 OR 灸法 OR 灸疗 OR 灸术 OR 温针 OR 针法 OR 三棱针 OR 皮肤针 OR 芒针 OR 眼针 OR 手针 OR 足针 OR 腕踝针 OR 平衡针 OR 揿针 OR 皮内针 OR 腹针 OR 舌针 OR 项针) OR R=(针刺 OR 电针 OR 针灸 OR 刺法 OR 经皮穴位电刺激 OR 体针 OR 头针 OR 毫针 OR 针刺治疗 OR 针灸疗法 OR 针法 OR 手针) AND (中风 OR 卒中 OR 脑梗死 OR 脑梗塞 OR 脑出血) AND (磁共振 OR 影像 OR fMRI OR MRI OR 灰质 OR 白质 OR 体素 OR 独立成分分析 OR ICA OR ROI OR 种子相关分析 OR ALFF OR 低频振幅 OR fALFF OR ReHo OR 局域一致性 OR 血氧水平依赖性 OR BOLD OR 基于体素的分析 OR 基于体素的形态测量 OR 皮质厚度)

**results：n=723**


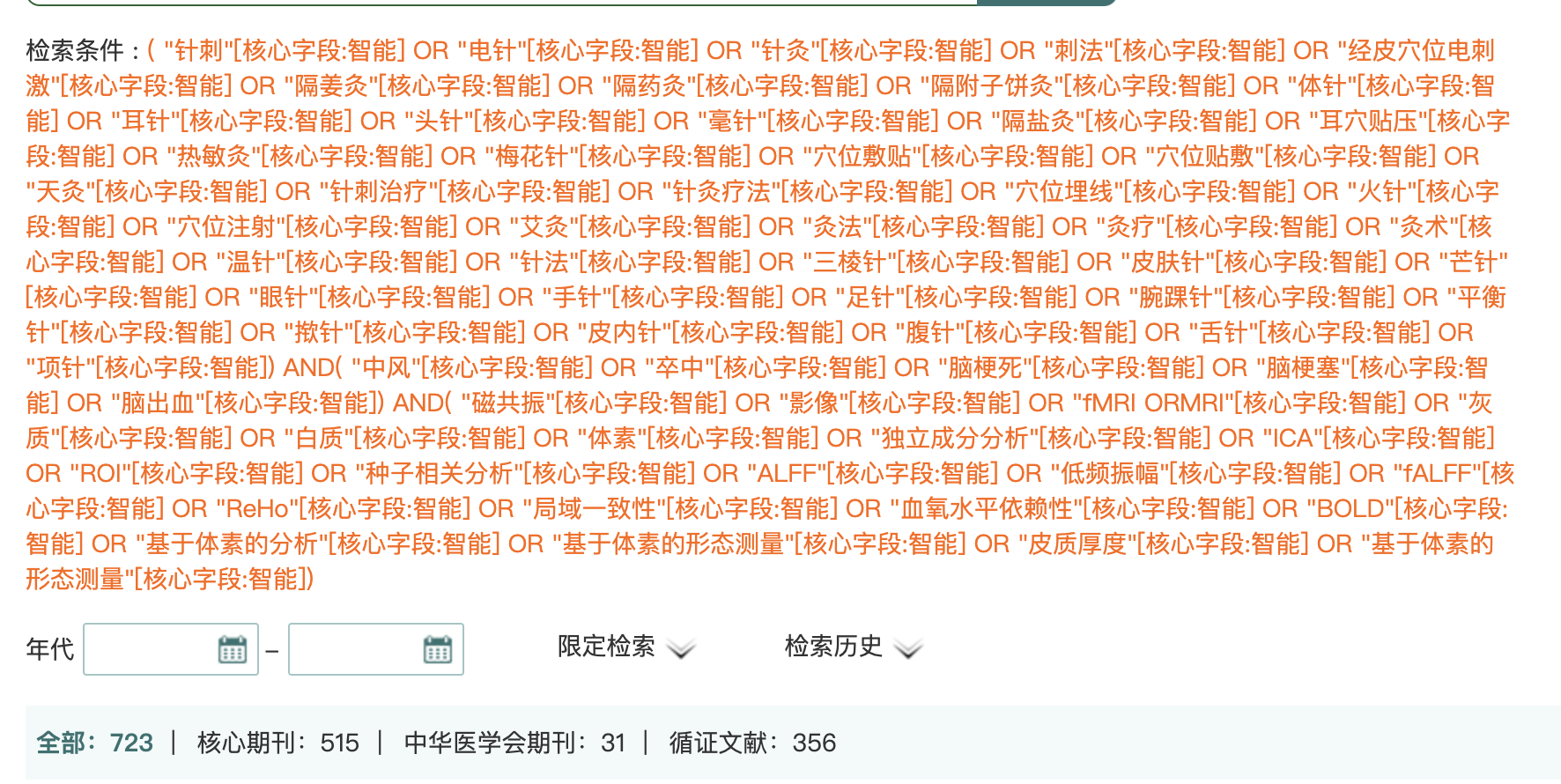

Supplement: Supplementary file 1 [file Data_Sheet_1.docx]
